# Influenza Virus Infection in Guinea Pigs Raised as Livestock, Ecuador

**DOI:** 10.3201/eid1807.111930

**Published:** 2012-07

**Authors:** Victor H. Leyva-Grado, Samira Mubareka, Florian Krammer, Washington B. Cárdenas, Peter Palese

**Affiliations:** Mount Sinai School of Medicine, New York, New York, USA (V.H. Leyva-Grado, F. Krammer, P. Palese);; Sunnybrook Health Sciences Centre and Research Institute, Toronto, Ontario, Canada (S. Mubareka); and; Escuela Superior Politécnica del Litoral (ESPOL), Guayaquil, Ecuador (W.B. Cárdenas)

**Keywords:** guinea pigs, livestock, domestic, influenza, virus, influenza A, influenza B, avian influenza, subtype H5N1, Ecuador, viruses

## Abstract

To determine whether guinea pigs are infected with influenza virus in nature, we conducted a serologic study in domestic guinea pigs in Ecuador. Detection of antibodies against influenza A and B raises the question about the role of guinea pigs in the ecology and epidemiology of influenza virus in the region.

Influenza A virus infection causes disease in humans and domestic animals, including pigs, horses, and chickens, and seasonal epidemics among humans in the Northern and Southern Hemispheres result in hospitalizations and deaths worldwide. Influenza A virus transmission studies are often conducted in laboratory guinea pigs (1,2) because the virus can efficiently spread from infected animals to naive guinea pigs by direct and indirect (short-range infectious aerosols) contact (3,4). However, whether guinea pigs are naturally infected with influenza virus outside the laboratory setting is not known.

In some regions of South America, guinea pigs are part of the traditional cuisine and are produced as livestock and sold commercially for human consumption. Guinea pigs are customarily raised on small rural farms in proximity to other livestock. Circulation of influenza virus in these populations has not been studied. Given the effect of influenza virus on human health and the susceptibility of guinea pigs to influenza virus infection in the laboratory, it is worthwhile to determine whether influenza virus can spread among guinea pigs in agricultural settings. As an initial step in this endeavor, we obtained serum samples from domestic guinea pigs in Ecuador and tested them for the presence of influenza antibodies to determine whether the guinea pigs had been infected with influenza virus.

## The Study

**Figure 1:**
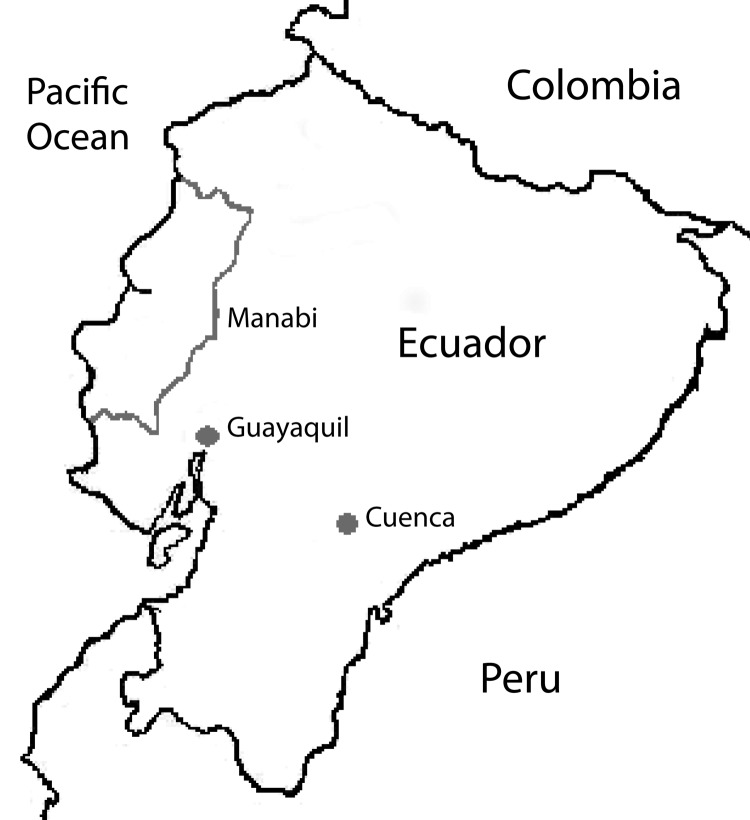
Three regions of Ecuador where guinea pig serum samples were obtained: Cuenca, Guayaquil, and Manabi. The country is bordered by Colombia to the north, Peru to the east and south, and the Pacific Ocean to the west. Cuenca is located in the Andes; the average annual mean temperature is 14.7°C, and the average annual relative humidity is 85%. Guayaquil is located at the head of the Gulf of Guayaquil; the mean temperature is 26.1°C, and relative humidity is 74%. The Manabí region is located on the Pacific Ocean coast; the mean temperature is 25.9°C, and relative humidity is 79%.

We obtained serum samples from 40 guinea pigs from 3 different regions of Ecuador (Figure 1), 20 from Cuenca and 10 each from Guayaquil and the Manabí region. Cuenca is located in the Andes region, 2,500 m above sea level; it is one of the main producers of guinea pig meat. Guayaquil, the most populated city in Ecuador, is located at the head of the Gulf of Guayaquil on the Pacific Ocean. The Manabí region is located in western Ecuador on the Pacific Ocean coast. Serum samples were collected from adult guinea pigs that we purchased from local farms (Cuenca), where they had been raised as livestock, or from live animal markets (Guayaquil and Manabí). The samples were collected by heart puncture under general anesthesia (combination of ketamine and xylazine). Animals were euthanized after samples were obtained.

For antigens in the serologic analyses, we used whole viruses and recombinant hemagglutinin, nucleoprotein, and neuraminidase (subtypes N1 and N2) proteins produced in our laboratory as described (5,6). The whole viruses were A/Brisbane/59/2007 (H1N1) (Brisbane07), A/New Caledonia/20/1999 (H1N1) (Newcal99), A/Wisconsin/67/2005 (H3N2) (Wisconsin05), A/Vietnam/1203/2004 (H5N1) (Vietnam04), and B/Yamagata/16/1988 (Yamagata88). The hemagglutinins were A/California/04/2009 (Cal09), NewCal99, Vietnam04, Wisconsin05, and Yamagata88. The nucleoproteins were Brisbane07, A/Puerto Rico/08/1934, and B/Florida/04/2006. The subtype N1 neuraminidases were Cal09 and Vietnam04; the N2 subtype was A/Hong Kong/1/1968.

ELISA was done as described (7), with slight modifications for the use of guinea pig serum. The cutoff for serum considered positive for influenza virus was the value of the negative control (naive guinea pig serum) +3 SD (from 3 repetitions). For Western blot (WB) analyses (8), serum samples were pooled into groups of 5 (groups 1–4, 5–6, and 7–8 were from Cuenca, Guayaquil, and Manabí region, respectively). Pooled samples were also used for the hemagglutination inhibition (HI) assay, as described (9), using influenza strains Brisbane07, Wisconsin05, and Vietnam04. As positive controls, we used serum from guinea pigs that we had infected with Cal09, Brisbane07, NewCal99, Wisconsin05, Vietnam04, or Yamagata88.

ELISA results for the 40 serum samples showed that 20, 18, and 14 were positive for influenza subtypes H1, H3, and H5, respectively (Table 1). The samples were also tested for the presence of antibodies against the influenza virus nucleoprotein: results for 29 were positive. Samples with positive results to >1 hemagglutinin antigens and to nucleoprotein were also analyzed for the presence of antibodies against proteins of the neuraminidase subtypes N1 (Cal09, Vietnam04) and N2 (influenza A/Hong Kong/1/1968 [H3N2]) (Table 1). Serum samples were also tested for the presence of influenza B virus by using whole virus, recombinant hemagglutinin, and recombinant nucleoprotein as described above. Samples from Cuenca showed the highest overall positivity to the 3 antigens (Table 2).

**Table 1:** ELISA results for the presence of influenza A virus antibodies in guinea pigs from different regions of Ecuador*

Region	No. (%) positive, by antigen†						
	H1	H3	H5	NP	N1 (Cal09)	N1 (Vietnam04)	N2
Cuenca, n = 20	11 (55)	12 (60)	6 (30)	16 (80)	8 (57)‡	8 (57)‡	10 (71)
Guayaquil, n = 10	8 (80)	5 (50)	8 (80)	10 (100)	8 (88)§	8 (88)§	8 (88)§
Manabí, n = 10	1 (10)	1 (10)	0	3 (30)	1 (50)¶	1 (50)¶	0¶
Total	20	18	14	29	17	17	18

*Samples were from adult guinea pigs raised as livestock on local farms (Cuenca) or from live animal markets (Guayaquil and Manabí). H1, recombinant hemagglutinin from A/California/04/2009 (Cal09); H3, recombinant hemagglutinin from A/Wisconsin/67/2005; H5, recombinant hemagglutinin from A/Vietnam/1203/2004 (Vietnam04); NP, recombinant nucleoprotein from A/Brisbane/10/2007; N1, recombinant neuraminidase from Cal09 or Vietnam04; N2, recombinant neuraminidase from A/Hong Kong/1/1968. †Serum samples were considered positive if absorbance (read at 405 nm) was higher than the value of the negative control (naive guinea pig serum) +3 SD. ‡Only 14 samples were tested. §Only 9 samples were tested. ¶Only 2 samples were tested.

**Table 2:** ELISA results for the presence of influenza B virus antibodies in guinea pigs from different regions of Ecuador*

Region	No. (%) positive, by antigen†		
	Whole virus (Yamagata88)	Recombinant hemagglutinin	Recombinant nucleoprotein
Cuenca, n = 20	17 (85)	18 (90)	18 (94)‡
Guayaquil, n = 10	8 (80)	9 (90)	9 (90)
Manabí, n =10	2 (20)	1 (1)	1 (10)
Total	27	28	28

*Samples were from adult guinea pigs raised as livestock on local farms (Cuenca) or from live animal markets (Guayaquil and Manabí). Whole virus, B/Yamagata/16/1988. Recombinant hemagglutinin was derived from B/Florida/04/2006; nucleoprotein was derived from B/Yamagata/16/1988. †Serum samples were considered positive if absorbance (read at 405 nm) was higher than the value of the negative control (naive guinea pig serum) + 3 SD. ‡Only 19 samples were tested.

**Figure 2:**
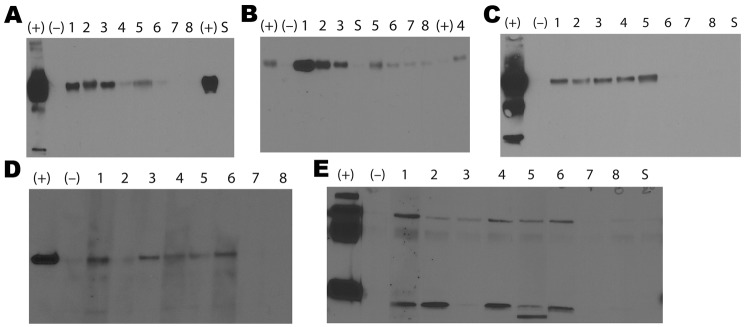
Results of Western blot analyses of pooled serum samples from adult guinea pigs in Ecuador. The guinea pigs were obtained from farms in Cuenca, where they had been raised as livestock, or from live animal markets in Guayaquil and Manabí. The results show different influenza antigens: recombinant hemagglutinin (rHA) A/New Caledonia/20/1999 (A); rHA A/Wisconsin/67/2005 (B); rHA A/Vietnam/1203/2004 (C); recombinant nucleoprotein A/Puerto Rico/08/1934 (D); and B virus (whole virus B/Yamagata/16/1988) (E). Molecular weights are shown at left. (+), serum from guinea pigs infected in the laboratory with influenza virus; (−), serum from naive guinea pig; lanes 1–4, pooled serum samples from Cuenca; lanes 5–6, pooled serum samples from Guayaquil; lanes 7–8, pooled serum samples from Manabí; S, secondary antibody only.

WB analysis of pooled samples from the 3 areas confirmed the ELISA results that showed antibodies against the hemagglutinin and nucleoprotein antigens; however, samples from Manabí region (7,8) showed immunoreactivity to the H3 antigen only (Figure 2). WB results for serum samples from Cuenca and Guayaquil were positive for influenza B virus, but results were not positive for samples from Manabí when whole virus was used as antigen (Figure 2). HI activity was observed in all pooled samples against all 3 viruses tested. Titers were highest in samples from Cuenca (320 for Brisbane07, 640 for Wisconsin05, and 80 for Vietnam04) and lowest for samples from Manabí (80 for Brisbane07 and 40 for Wisconsin05 and Vietnam04). Titers for samples from Guayaquil were 160 for Brisbane07 and 80 for Wisconsin05 and Vietnam04.

## Conclusions

We showed the presence of influenza virus antibodies with HI activity to different virus subtypes in guinea pigs marketed through local farms and live animal markets in different regions of Ecuador. Seroprevalence was similar for influenza A virus subtypes H1 (50%) and H3 (45%). A study of influenza among humans in Ecuador also showed similar seroprevalences for subtypes H1 (5.1%) and H3 (5.5%) (10). This10-fold difference between seroprevalence rates among humans and the guinea pigs in our study may be explained by animal husbandry practices that facilitate influenza transmission (e.g., the crowding of animals in cages).

The presence of antibodies to influenza B virus in guinea pigs is notable because infection with this virus is thought to be restricted to humans (11). Recent studies in our laboratory demonstrated that guinea pigs can also be infected with influenza B viruses and that they readily transmit the virus to naive guinea pigs (12). These studies support our finding that influenza B virus has the potential to be transmitted from humans to other species. Further studies are needed to isolate and characterize the type B influenza virus present in the population of guinea pigs to determine if there has been an adaptation to the new host or if the guinea pig is only a transient reservoir for the human virus.

We found that several guinea pigs had antibodies against influenza A (H5). Ecuador is purported to be free of avian influenza (13); however, reports from other countries in South America demonstrate the presence of avian subtypes H3, H5N2, H7N3, and H13N9 in wild birds (14). In addition, chickens, turkeys, and guinea pigs that are later sold in local farmers’ markets are raised together by some families, thereby facilitating contact of guinea pigs with avian influenza viruses. We tested only for seroreactivity to the H5 hemagglutinin and the N1 neuraminidase; therefore, further studies are needed to determine whether different avian origin influenza viruses are present in the guinea pig population.

We did not determine whether guinea pigs are an incidental host for influenza virus infection or, if instead, the virus has been adapted to these animals or if guinea pigs are a natural reservoir for some influenza viruses. To this end, virus isolation and characterization would be necessary to determine the virus strains circulating in this population. In the laboratory, guinea pigs are infected and efficiently transmit influenza viruses to naive hosts without showing any overt clinical signs of disease (1). Therefore, further studies are needed to address the specific role of guinea pigs raised as livestock in the ecology and epidemiology of influenza viruses in the region.
